# Metabolomic Changes in Major Depressive Disorder Adolescent Females with or without Suicide Attempts

**DOI:** 10.2174/1570159X23666250122093451

**Published:** 2025-01-22

**Authors:** Wei-Xi Deng, Xiao-Bo Liu, Tian Guo, Li-Fei Shang, Yi Li, Kuan Zeng, Jing-Yi Long

**Affiliations:** 1 Department of Radiology, Wuhan Mental Health Center, Wuhan, China;; 2 Affiliated Wuhan Mental Health Center, Tongji Medical College of Huazhong University of Science and Technology, Wuhan, China;; 3 Department of Psychiatry, The Second Affiliated Hospital and Yuying Children's Hospital of Wenzhou Medical University, Wenzhou, China

**Keywords:** Major depressive disorder, thyroid hormones, lipid metabolism, magnetic resonance spectroscopy, suicidal ideation, brain metabolism-related factors

## Abstract

**Background:**

The incidence of Major Depressive Disorder (MDD) is high among adolescent females, and MDD is often accompanied by suicide attempts (SAs), which have a serious negative impact on health. However, changes in lipids, thyroid hormone, and brain metabolism among female adolescents with MDD and the relationships between these three markers and MDD with SA have yet to be elucidated.

**Methods:**

This study enrolled 71 MDD patients with SA (MDD+SA), 66 MDD patients without SA (MDD-SA), and 47 healthy controls (HCs). We analysed the lipid and thyroid hormone levels and magnetic resonance spectroscopy results of the subjects.

**Results:**

Low levels of social support, high levels of life stress, and high levels of suicidal ideation (SI) were risk factors for SA. In MDD patients, 1) thyroid stimulating hormone was positively correlated with triglyceride (TG) and N-acetyl aspartic acid (NAA)/creatinine in the prefrontal cortex (PFC) and negatively correlated with high-density lipoprotein and the choline/creatinine in the thalamus; 2) free thyroxine was negatively correlated with the choline/creatinine in the thalamus; 3) total cholesterol, TG, low-density lipoprotein, and choline/NAA in the PFC were positively correlated with the severity of SI and suicide risk; and 4) NAA/creatinine in the thalamus was negatively correlated with the severity of SI and suicide risk.

**Conclusion:**

In female adolescents with MDD, there are significant synergistic changes in lipids, thyroid hormones, and brain metabolism-related factors, and the changes in these indicators may be related to the pathological mechanism of SA.

## 1 INTRODUCTION

Major depressive disorder (MDD) is characterized by a persistent low mood and is often accompanied by cognitive impairment [1, 2]. There was a 14% increase in MDD cases among adolescents worldwide between 2011 and 2020 [3]. Among adolescents aged 10-19 years, the prevalence of MDD is approximately 34%, and it is twice as common in females as in males. Among MDD patients, 10.8% have attempted suicide (SA) [4]. In the study by Pompili *et al*. [5], patients with symptoms of severe depression had a higher risk of suicide, and these patients were more frequently female. Suicide is a common problem in the global mental health field; the incidence of suicide continues to increase annually [6]. Specifically, suicide has become the fourth leading cause of death among adolescents worldwide [7]. The global incidence of suicide is approximately 12-20% [8], and the prevalence of SA in women is higher than that in men [9]. Adolescents are in the stage of rapid physical and mental development, experience large emotional fluctuations, and are prone to negative thoughts when they encounter pressures [10, 11]. Female adolescents may have a higher affective bias to negative stimuli than male adolescents and thus are prone to extreme behaviors [12]. Therefore, special attention should be given to recognizing SA in female adolescents with MDD [13, 14].

The neuropathological mechanisms of MDD in adolescents with a history of SA have not been fully elucidated, but several studies have suggested that thyroid and blood lipids play indispensable roles in neuronal function related to cognitive and emotional regulation [15-18]. Thyroid hormones may be associated with, or even predict, a suicide attempt (SA) [19]. Mildly increased thyroid stimulating hormone (TSH) levels are often found in MDD patients and are considered related to the suppression of autoimmune mechanisms and hypothalamic-pituitary-thyroid (HPT) axis abnormalities caused by MDD [20, 21]. The TSH level in first-episode, unmedicated MDD patients with SA was greater than that in MDD patients without SA, indicating that TSH may be a characteristic metabolic index of SA [22].

Obesity is considered an independent risk factor for MDD in adolescents (especially females) [23]. At present, the correlation between lipid metabolism and SA has not been completely resolved. Dyslipidaemia, especially low total cholesterol (TC), increases the risk of SA by changing the membrane structure and affecting receptors such as five-hydroxytryptamine (5-HT) [24-26]. It has also been shown that SA is associated with increased TC, increased low-density lipoprotein (LDL), and decreased high-density lipoprotein (HDL) [27]. In female adolescents with MDD, there is a U-shaped correlation between body mass index (BMI) and SA, meaning both high and low BMI are associated with a greater risk of SAs [28, 29]. Therefore, further exploration of the relationship between thyroid function, blood lipids, and suicide risk in MDD patients with a history of SA could help in further understanding the complex metabolic mechanisms involved and positively guide the recognition of early interventions for adolescents at risk for suicide.

Magnetic resonance spectroscopy (MRS) can be used to noninvasively and quantitatively detect brain metabolic changes and has unique advantages in the study of neurometabolism in MDD patients [30,_ 31]. Previous studies have shown that MDD patients have reduced N-acetyl aspartic acid (NAA) concentrations in the bilateral prefrontal cortex (PFC) and a reduced NAA/creatine (Cr) ratio, suggesting that neuronal activity, integrity, and function are impaired in the PFC [32, 33]. A longitudinal study using theta-burst stimulation (TBS) to treat MDD revealed that the choline (Cho)/NAA ratio in the anterior cingulate cortex (ACC) increased and was a potential mechanism of improvement in MDD symptoms [34].

In general, most relevant existing studies have investigated the risk factors for and pathogenic mechanisms of SA in MDD patients from the perspective of a single discipline/clinical indicator. There is a lack of multidimensional metabolism studies on thyroid function, blood lipids, and the MRS of SAs in female adolescents with MDD. Therefore, in this study, the changes in thyroid hormones, blood lipids, and brain metabolism-related factors in female adolescents with MDD were analysed, and synergistic changes among the three factors were observed to reveal the metabolic, neural mechanism related to SA.

## 2 MATERIALS AND METHODS

### 2.1 Study Subjects

Female adolescents with MDD in the outpatient and inpatient departments of Wuhan Mental Health Centre (WMHC) from October 2021 to October 2023 were enrolled. Healthy controls (HCs) were recruited by advertisement. The inclusion criteria for the MDD group were as follows: 1) met the diagnostic criteria for MDD in the Diagnostic and Statistical Manual of Mental Disorders 5^th^ edition (DSM-5); 2) had residual MDD symptoms (score ≥ 19) on the Children's Depression Inventory (CDI) assessment, regardless of previous treatment; 3) were female; 4) were of Han nationality; 5) were aged 12-17 years; 6) were right-handed; and 7) had at least an elementary school education level. The exclusion criteria for the MDD group were as follows: 1) previous or current presence of other mental disorders in the DSM-5 and the presence of secondary depression caused by substance dependence or abuse; 2) previous or current presence of severe physical diseases of other systems; 3) history of electroconvulsive therapy (ECT) within 1 month prior to enrolment; and 4) family history of mental disorders. The inclusion criteria for the HC group were as follows: 1) no mental or nervous system diseases; 2) total CDI score < 19 points; and 3) female sex and right-handedness. The exclusion criteria for the HC group were as follows: 1) met the diagnostic criteria for MDD in the DSM-5; 2) previous or current presence of other mental disorders in the DSM-5; 3) substance abuse; 4) previous or current presence of severe physical diseases of other systems; and 5) family history of mental disorders.

### 2.2 Demographic Information Collection

After enrollment, the basic information on the subjects was collected *via* questionnaires. The main information collected included age, nationality, years of education, disease course, current academic performance level, monthly disposable income, family status, relationship with close contacts, satisfaction with school, and description of self-health status.

### 2.3 Clinical Data Collection

All the patients were assessed for psychiatric diagnoses according to the DSM-5 and underwent a semi-structured interview with two psychiatrists who have more than 5 years of work experience and consistency training. The interviews lasted approximately 30 minutes to 1 hour. The clinical data of all the subjects were collected from medical records, and each participant completed the Beck Scale for Suicide Ideation-Chinese Version (BSI-CV), the Adolescent Self-Rating Life Events Checklist (ASLEC) and the Adolescent Social Support Scale (ASSS). In addition, MDD patients were evaluated using the Self-Rating Anxiety Scale (SAS) and Self-Rating Depression Scale (SDS). According to the Columbia Classification Algorithm for Suicide Assessment (C-CASA), SA was defined as: “there is a certain degree of willingness to end one’s life, and actions have been taken to achieve death, but without success” [35]. The medical history of all patients was reviewed, and the patients were divided into the MDD patients with SA (MDD+SA) group and the MDD patients without SA (MDD-SA) group based on whether there was at least one SA in the past month.

### 2.4 Blood Specimen Collection

Venous plasma was collected through venepuncture of a cubital vein in the arm at 7-10 a.m. on the next day after patient enrolment. Participants were asked to fast for 10 hours before the sample was obtained and to not smoke, drink, or use any illegal drugs within 24 hours before the visit. Within 20 min after the sample was obtained, it was sent to the hospital haematology laboratory. Serum was isolated by centrifugation at 3,000 rpm for 15 min. An automatic chemiluminescence analyser was used to detect TSH, free triiodothyronine (FT3), and free thyroxine (FT4). The triglyceride (TG), TC, HDL, and LDL levels were measured with an AU680 Beckman Curl automatic biochemical analyser.

### 2.5 MRS Data Acquisition

Magnetic resonance imaging (MRI) scanning was performed within 48 hours after completion of the psychological scale. MRS images were acquired at the Department of Medical Imaging of WMHC using a 3.0 T MRI scanner (Ingenia, Philips, Netherlands) and a Head/Neck 20-channel coil (Philips, Netherlands), and the parameters were as follows: point-resolved spectroscopy (PRESSP); single-voxel spectroscopy; TR=2000 ms; TE=144 ms; Min.TR/TE (ms)=838/53, and number of signals averaged=128. The built-in software of the MRI scanner automatically performed baseline calibration, signal averaging, and metabolite identification and measured the peak areas of NAA, Cho, and Cr in the ACC, PFC, and thalamus. The NAA peak area was 2.0 ppm, the Cho peak area was 3.21 ppm, and the Cr peak area was 3.02 ppm.

### 2.6 Statistical Analysis

All clinical data analyses were performed using SPSS 29.0 (https://www.statistical-analysis.top/SPSS). Normally distributed data are expressed as means ± SDs (±s); comparisons were performed by two-sided *t*-tests and one-way analysis of variance (ANOVA). Nonnormally distributed data are expressed as the median and interquartile range [M (P25, P75)]; comparisons were performed by the Mann‒Whitney U test and the Kruskal-Wallis H test. The counting data were analysed using the chi-square test and post hoc test. Spearman’s correlation analysis was used to evaluate the correlation between metabolic indicators and clinical psychological characteristics in the MDD group. A difference was considered statistically significant when *p <* 0.05.

## 3 RESULTS

### 3.1 Overview of the Demographic and Clinical Data

A total of 71 patients (median age, 15 years) were enrolled in the MDD+SA group; the disease course ranged from 1-54 months, with a median of 23 months. There were 66 patients (median age, 15 years) in the MDD-SA group; the disease course ranged from 1-49 months, with a median age of 14.5 months. There were 47 individuals (median age, 14 years) in the HC group. There was no difference in disease duration between the two groups, and there were no significant differences in age, years of education, current academic performance level, monthly disposable income, smoking history, or alcohol consumption history among the three groups (*p >* 0.05) (Table **1**) (refer to Table S**1** for details).

### 3.2 Comparison of Clinical Characteristics among the Three Groups

1) Relationships with close contacts, satisfaction with school, and description of self-health status in the MDD+SA group and MDD-SA group were significantly worse than those in the HC group. 2) The CDI scores were significantly different among the three groups, with the MDD+SA group having the highest scores, followed by the MDD-SA group and the HC group. 3) The scores for suicide ideation (SI), SI in the past week, SI at the most depressed time, suicide risk, suicide risk in the past week, and suicide risk at the most depressed time were significantly different among the three groups; the scores for SA-related indicators were highest in the MDD+SA group, followed by the MDD-SA group and the HC group. 4) The SAS and SDS scores and severity of SI in the MDD+SA group were significantly higher than those in the MDD-SA group. 5) For the total scores for life events, interpersonal relationship factors, health adaptation factors, penalty factors, and loss factors, the scores in the MDD+SA group were significantly greater than those in the MDD-SA and HC groups; the study stress factor scores were highest in the MDD+SA group, followed by the MDD-SA group and the HC group. 6) The total scores for social support, subjective support, and objective support were lower in the MDD+SA group than in the MDD-SA and HC groups; furthermore, the scores for support utilization were lowest in the MDD+SA group, followed by the MDD-SA group and the HC group. These results were all statistically significant (*p <* 0.05) (Table **1**) (refer to Table **S1** for details).

**Fig. (1):**
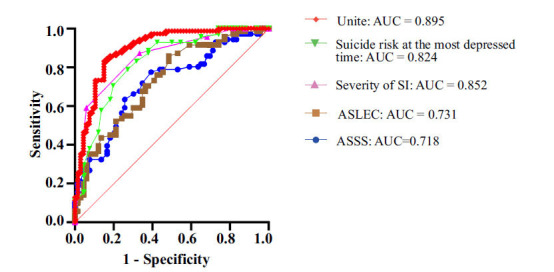
Predictors of incident suicide attempts among adolescent MDD patients.

Logistic regression analysis was performed with SA as the dependent variable. The results revealed that a high risk of suicide at the most depressed time (*p =* 0.025), a high total score for life events (*p =* 0.008), and severity of SI (*p <* 0.001) were risk factors for SA in female adolescents with MDD; a high social support score was a protective factor against SA in female adolescents with MDD (*p =* 0.035). To evaluate the predictive effect of the above four influencing factors on the occurrence of suicide attempts in depressed patients, the ROC curve was further drawn. The area under the curve (AUC) for the severity of SI, the suicide risk at the most depressed time, life events, and the total score for social support were 0.852, 0.824, 0.731, and 0.718, respectively. After combining the above factors, the AUC was 0.895, which was significantly higher than the AUC of any single influencing factor (*p <* 0.05). According to the Youden index (YI), the optimal sensitivity for the combined factors was 0.679, the specificity was 0.848, and the sensitivity was 0.831 (Fig. **1**) (refer to Table **S2** for details).

### 3.3 Thyroid Hormones

A total of 56 MDD+SA patients and 45 MDD-SA patients completed thyroid function tests. Eight patients in the MDD+SA group had subclinical hypothyroidism (SCH). In the MDD-SA group, 2 patients had SCH. The median TSH, FT3, and FT4 levels in the MDD+SA group were 2.47, 2.95, and 1.19, respectively, and the median TSH, FT3, and FT4 levels in the MDD-SA group were 2.31, 2.86, and 1.18, respectively. The thyroid levels in the MDD+SA group were greater than those in the MDD-SA group, but the difference was not statistically significant (*p >* 0.05) (Table **2**).

### 3.4 Lipid Metabolism

The lipid levels of 59 MDD+SA patients and 51 MDD-SA patients were analysed. The median BMIs of the MDD+SA and MDD-SA groups were 20.14 and 19.49, respectively. Thirteen patients in the MDD+SA group had abnormal blood lipid levels, and 10 patients in the MDD-SA group had abnormal blood lipid levels. The blood lipid levels (except HDL) in the MDD+SA group were greater than those in the MDD-SA group, but the differences were not statistically significant (*p >* 0.05) (Table **2**).

### 3.5 MRS

**Fig. (2):**
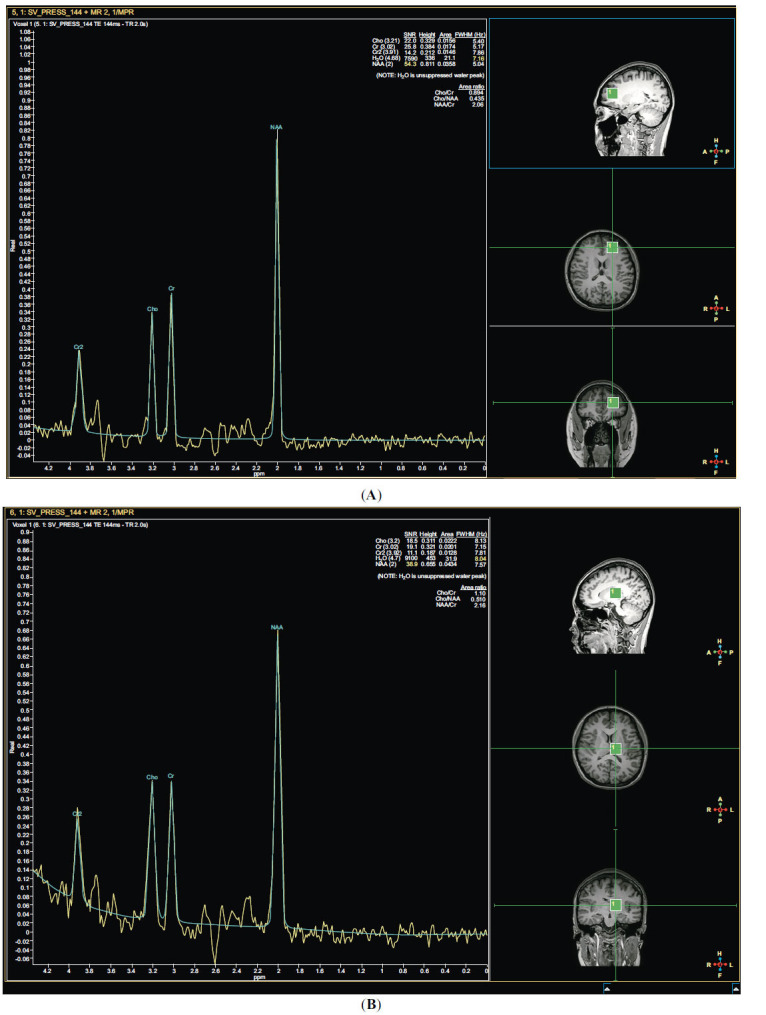
(**A**) Neurochemical metabolic indicators of the PFC on MRS. (**B**) Neurochemical metabolic indicators of the thalamus on MRS.

MRS was completed for 31 patients in the MDD+SA group, 22 patients in the MDD-SA group, and 20 individuals in the HC group. The Cho/Cr ratio in the PFC was significantly greater in the MDD+SA group than in the HC group; the NAA/Cr ratio was significantly greater in the MDD+SA and MDD-SA groups than in the HC group; and the NAA/Cr ratio in the thalamus was significantly lower in the MDD+SA group than in the MDD-SA group. These results were statistically significant (*p <* 0.05) (Table **2**, Fig. **2**) (refer to Table **S3** for details).

### 3.6 Correlations between Metabolites and Psychological Traits in MDD Patients

**Fig. (3):**
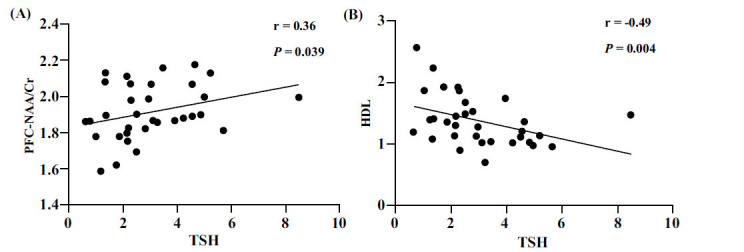
(**A**) TSH was significantly positively correlated with the NAA/Cr ratio in the PFC; (**B**) TSH was significantly negatively correlated with HDL levels.

**Fig. (4):**
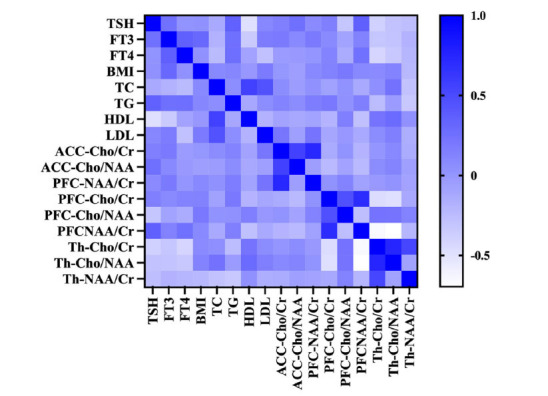
Correlation plots between metabolites in MDD patients.

A total of 19 MDD+SA patients and 14 MDD-SA patients underwent thyroid function, blood lipid, and MRS tests. The results showed that 1) TSH was significantly positively correlated with TG and the NAA/Cr ratio in the PFC and negatively correlated with HDL level and the Cho/Cr ratio in the thalamus; 2) FT3 was negatively correlated with HDL level; and 3) FT4 was negatively correlated with the Cho/Cr ratio in the thalamus. 4) TC, TG, LDL, and the Cho/NAA ratio in the PFC were positively correlated with the severity of SI and suicide risk. 5) The NAA/Cr ratio in the thalamus was negatively correlated with the severity of SI and suicide risk. These results were all statistically significant (*p <* 0.05) (Table **3**, Figs. **3** and **4**) (refer to Table **S4** for details).

## 4 DISCUSSION

### 4.1 Social and Psychological Factors

In this study, scores for social support factors were significantly lower in the MDD+SA group than in the other two groups. Scores for depression, anxiety, and life event factors were significantly greater in the MDD+SA group than in the other two groups. The possible reasons for these differences are a lack of support from people around them [36], life stressors [37], a tense family atmosphere [38], and childhood trauma history [39], all of which can lead to reduced adaptability and increased negative bias cognition among adolescents. Individuals with high levels of depression and anxiety are prone to high rumination and highly sensitive stress perception [40]. In particular, for Chinese teenagers aged 13-17 years who are in middle school, heavy schoolwork loads, fierce competition, and continuous mental stress can all cause/aggravate negative conditions such as loneliness, loss, depression, and even SA [41, 42]. In this study, in addition to the significant correlation between SA and negative life events and social support, the severity of SI and suicide risk (especially during the most depressed time) effectively predicted SA [39, 43, 44]. Therefore, we believe that regularly evaluating recent thoughts on suicide is helpful for the early identification of the risk of SA in adolescents with MDD and that early psychological intervention could help reduce the suicide rate in this population.

### 4.2 Thyroid Hormone Metabolism

The detection rate of SCH in female adolescents with MDD was 14.29%, which is higher than the 3.1% prevalence among healthy adolescents reported in previous studies [45]. The risk of developing MDD in adult SCH patients is 2.5 times greater than that in healthy individuals [46]. Several scholars have reported that the detection rate of SCH in MDD patients is greater than that in the healthy population [47, 48] and that the increase in TSH secretion might be due to abnormal functioning of the pituitary gland [49-51]. More importantly, the interaction between the HPT axis, norepinephrine, and 5-hydroxytryptamine (5-HT) system is a potential mechanism underlying the risk of SA in patients with MDD and SCH [52-54], and thyroid hormones play important roles in this process [55]. This study suggested that the impact of abnormal thyroid function on adolescents with MDD should not be ignored. Therefore, early detection and dynamic observation of thyroid function are recommended for adolescents with MDD.

### 4.3 Lipid Metabolism

In females with MDD, BMI and social support were negatively correlated, and BMI and suicide risk were positively correlated. This finding is consistent with the results of the present study [56]. In female MDD patients, due to the stigmatization of their body shape, high perception of body image [57, 58], and reduced response to drug treatment in overweight or obese individuals [59], the risk of SA increases. This study also showed that higher TC, TG, and LDL levels were associated with higher SI and suicide risk, further supporting previous studies showing that changes in blood lipid levels may have a potential relationship with the pathological mechanism of suicide risk in MDD patients [60]. In this study, there were no significant differences in blood lipids in the MDD groups, possibly because the subjects of this study were female adolescents. Given that the probability of developing lipid metabolism abnormalities in adolescents is relatively lower than that in adults, in future studies, we will longitudinally follow up with the subjects to observe changes in lipid metabolism and the intrinsic association with suicide.

### 4.4 Neurobiochemistry

NAA content can indicate neuronal density [61]; Cr has been used as an internal standard for proton magnetic resonance spectroscopy (^1^H-MRS) [62]; and Cho reflects the level of phospholipid metabolism [63] and has been shown to be positively correlated with the severity of MDD [34, 64]. All three parameters are important biochemical indicators of brain metabolism [65, 66]. The results regarding NAA/Cr and Cho/Cr in the PFC contradict current studies [67-69]. We suggest that, first, adolescence is a critical period of neurodevelopment in which neural circuits are immature, and there may be some degree of compensatory capacity [70]. The process of MDD is dynamic and dependent on developmental stages [71]. Finally, this may also be due to the heterogeneity of the patient sample; our sample was composed of female adolescents. As a key area of the brain that processes emotional and cognitive information, the PFC has been extensively confirmed as a core component in the pathogenesis of MDD and is closely related to SA [72]. The results of this study suggested that neurometabolic changes in the PFC are an important neural basis in MDD patients and that metabolic changes in the PFC still need to be considered in adolescents [73]. Previous studies have shown a possible relationship between a reduction in cerebral NAA/Cr and higher levels of cognitive impairment as well as greater depression severity in MDD patients, which supports our finding that the NAA/Cr ratio in the thalamus was lower in MDD+SA than in MDD-SA [74-76]. Therefore, the NAA/Cr ratio in the thalamus deserves more attention in future studies.

### 4.5 Thyroid Hormone-lipid-neurochemical Metabolism Interactions

In terms of the synergistic changes in metabolic indicators, TSH is negatively correlated with HDL levels and positively correlated with TG levels, which may be related to the increase in TC, TG, and LDL levels in the hepatic endoplasmic reticulum (ER) induced by TSH and hepatic fat deposition [77] and the positive regulation of FT3 concentration, which indirectly affects HDL [78]. Second, the PFC Cho/Cr ratio was positively correlated with the TSH level. Previous studies have shown that MDD patients may have abnormal neurometabolism and thyroid function in the early stage and that the NAA/Cr ratio in the PFC is positively correlated with the TSH level [79]. These findings are highly consistent with our results. Finally, FT4 and TSH levels were negatively correlated with Cho/Cr in the thalamus. In similar reports, a high Cho concentration has been shown to be a marker for assessing neural structural integrity and functional abnormalities [80]. In addition, previous studies have confirmed that abnormal thyroid function (especially FT4 levels) is associated with neurometabolic abnormalities in the PFC-thalamus circuit [81]_. The Cho/NAA ratio in the PFC was positively correlated with the SI at the most depressed time. Therefore, we speculate that thalamic neuronal dysfunction in MDD patients may be closely related to abnormal FT4 levels. A larger sample size and longitudinal observation are needed in future related studies. Interestingly, the thalamic NAA/Cr ratio was negatively correlated with the severity of SA and the severity of suicide risk. This ratio may be a relevant indicator for the early diagnosis of SA in female adolescents, but further exploration with a larger sample size is needed. To our knowledge, this is a meaningful study in which synergistic changes in thalamic and PFC neurometabolism, thyroid hormone levels, and blood lipid metabolism have been observed in MDD patients. Our findings provide new evidence for a better understanding of synergistic changes in neurobiochemistry, thyroid function, and lipid metabolism.

### 4.6 Limitations

First, this was a case-control study, and therefore, it is difficult to ascertain the causal relationships between SA and thyroid dysfunction, blood lipid levels, and neurobiochemistry. In the future, we will carry out longitudinal studies to analyse the causal relationships and dynamic changes among the three factors. Second, this study did not compare first-episode depressed patients with chronically depressed patients, and further subgrouping and comparisons of acute and chronic patients are needed in the future. Third, blood specimens and MRS data could not be collected from some subjects. In future studies, we will recruit more participants and comprehensively analyse metabolomic changes in MDD+SA patients.

## CONCLUSION

As a viable choice for escaping unbearable negative emotions, it is important to understand the mechanisms underlying SA [82]. This study confirmed multiple differences in metabolic indexes among the MDD+SA, MDD-SA, and HC groups. Moreover, there were significant synergistic changes among the three types of factors, and some single/ comprehensive indicators may be potential mechanisms or related to the appearance and details of SA, especially the thalamic NAA/Cr ratio. The findings of this study provide new evidence for understanding MDD and suicide-related metabolic-neural mechanisms.

## Figures and Tables

**Table 1:** Clinical and demographic characteristics of the three groups [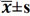/M (P25, P75)] or [n (%)].

**Variable**		**MDD + SA (n=71)**	**MDD-SA (n=66)**	**HC (n=47)**	**H/Z/χ2**	***p***	**Contrast**
**Sociodemographic**							
Age (years)		15(13,16)	15(13.75,16)	14(13,16)	3.594	0.166^a^	-
Education (years)		8(7,9)	8(7,10)	7(6,10)	2.836	0.242^a^	-
Disease course (months)		23(7,30)	14.5(5,26.5)	-	1.639	0.101^b^	-
Family status	Harmony	4(5.63%)	8(12.12%)	14(29.79%)	26.154	<0.001^c^	-
	Stable	32(45.07%)	42(63.64%)	27(57.45%)	-	-	-
	General	24(33.80%)	10(15.15%)	4(8.51%)	-	-	-
	Bad	11(15.49%)	6(9.09%)	2(4.26%)	-	-	-
Relationship with close contacts	Satisfactory	2(2.82%)	6(9.09%)	7(14.89%)	34.660	<0.001^d^	-
	Fairly satisfied	11(15.49%)	13(19.7%)	25(53.19%)	-	-	-
	General	38(53.52%)	33(50.00%)	13(27.66%)	-	-	-
	Less satisfied	12(16.90%)	10(15.15%)	2(4.26%)	-	-	-
	Unsatisfied	8(11.27%)	4(6.06%)	0(0.00%)	-	-	-
**Clinical**							
CDI		34.35±7.70	27(19.75,33)	15(8,17)	116.828	<0.001^a^	S>NS>N
BSI-CV		24(21,27)	20(16,24)	14(11,20)	60.663	<0.001^a^	S>NS>N
Severity of SI	Specific plans	42(59.15%)	4(6.06%)	-	60.841	<0.001^d^	-
ASLEC		62.96±19.61	46.26±19.11	35(20,69)	27.986	<0.001^a^	S>NS, N
ASSS		43.45±14.57	53(44.75,65)	64(52,78)	35.948	<0.001^a^	S<NS, N

**Note**: a. Kruskal-Wallis H Test; b. Mann-Whitney U test; c. Pearson's chi-squared test; d. Fisher's exact test.

**Abbreviations:** Healthy control (HC or N), Depression with suicide attempt (MDD+SA(S)), Depression without suicide attempt (MDD-SA(NS)), Children's Depression Inventory (CDI), Scale for Suicide Ideation-Chinese Version (BSI-CV), Severity of suicidal ideation (Severity of SI), Adolescent Self-Rating Life Events Check List (ASLEC), Adolescent Social Support Scale (ASSS).

**Table 2:** Comparison of metabolic indexes [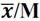].

**Variable**	**MDD+SA**	**MDD-SA**	**HC**	**Z/*t*/χ2/H**	***p*-value**	**Contrast**
**Sample Size**	56	45	-	-	-	-
TSH (uIU/ml)	2.47	2.31	-	0.307	0.758^a^	-
FT3(pg/ml)	2.95	2.86	-	1.787	0.074^a^	-
FT4(ng/dL)	1.19	1.18	-	0.673	0.501^a^	-
**Sample Size**	59	51	-	-	-	-
TC (mmol/L)	4.01	3.90	-	-0.778	0.433^b^	-
TC (mmol/L)	0.95	0.91	-	0.707	0.479^a^	-
HDL (mmol/L)	1.21	1.26	-	-0.492	0.623^a^	-
LDL (mmol/L)	1.96	1.75	-	1.037	0.300^a^	-
BMI	20.14	19.49	-	0.986	0.324^a^	-
**Sample Size**	31	22	20	-	-	-
ACC-Cho/Cr	1.01	1.06	1.02	0.388	0.824^d^	-
ACC-Cho/NAA	0.63	0.65	0.66	0.987	0.611^d^	-
ACC-NAA/Cr	1.57	1.65	1.54	2.820	0.244^d^	-
PFC-Cho/Cr	0.96	0.93	0.89	7.331	0.026^d^	S>N
PFC-Cho/NAA	0.50	0.47	0.57	2.426	0.297^d^	-
PFC-NAA/Cr	1.96	1.92	1.68	7.401	0.001^e^	S, NS>N
Thalamus-Cho/Cr	0.95	0.98	0.97	0.477	0.623^e^	-
Thalamus-Cho/NAA	0.50	0.48	0.48	1.588	0.452^c^	-
Thalamus-NAA/Cr	1.91	2.00	1.98	6.773	0.034^c^	S<NS

**Note**: a. Mann-Whitney U test, b. two-sided *t* test, c. Fisher's exact test, d. Kruskal-Wallis H Test, e. One-way analysis of variance.

**Abbreviations:** Thyroid stimulating hormone (TSH), Free triiodothyronine (FT3), Free thyroxine (FT4), Total cholesterol (TC), Triglyceride (TG), High-density lipoprotein (HDL), Low-density Lipoprotein (LDL), Body mass index (BMI), Anterior cingulate cortex (ACC), Choline/creatine (Cho/Cr), Choline/N-acetyl aspartic acid (Cho/NAA), N-acetyl aspartic acid/creatine (NAA/Cr), Prefrontal cortex (PFC).

**Table 3:** Correlation analysis of clinical features and metabolic indexes in MDD patients.

-	**BMI**	**TC**	**TG**	**HDL**	**LDL**	**ACC-Cho/NAA**	**PFC-Cho/NAA**	**PFC-NAA/Cr**	**Th-Cho/Cr**	**Th- NAA/Cr**
BSI-CV	0.42*	0.36*	0.16	0.21	0.31	-0.08	0.28	-0.26	-0.06	-0.41*
SI in the past week	0.34	0.32	0.12	0.08	0.51**	-0.14	0.04	-0.30	-0.04	-0.43*
SI at the most depressed time	0.42*	0.30	0.15	0.24	-0.04	-0.04	0.39*	-0.12	-0.08	-0.33
SR	0.43*	0.37*	0.35*	0.14	0.35*	-0.02	0.08	-0.23	-0.03	-0.46**
SR in the past week	0.43*	0.25	0.24	0.05	0.40*	-0.03	0.12	-0.25	-0.02	-0.42*
SR at the most depressed time	0.36*	0.47**	0.41*	0.19	0.19	-0.04	-0.01	-0.08	-0.05	-0.43*
The intensity of SI	0.35*	0.37*	0.32	0.09	0.20	-0.04	0.25	-0.17	-0.07	-0.35*
ASSS	-0.52**	-0.12	-0.20	-0.03	-0.24	0.38*	-0.25	-0.02	-0.04	0.23
TSH	0.03	-0.13	0.35*	-0.49**	0.10	0.25	-0.32	0.36*	-0.39*	-0.27
FT3	0.31	-0.18	0.25	-0.35*	0.18	0.09	-0.08	0.13	-0.31	-0.19
FT4	0.03	-0.24	0.27	-0.09	-0.29	-0.01	-0.15	0.24	-0.42*	-0.21

**Note: ** *p < 0.05, **p < 0.01.

**Abbreviations:** Scale for Suicide Ideation-Chinese Version (BSI-CV), Suicidal ideation in the past week (SI in the past week), Suicidal ideation at the most depressed time (SI at the most depressed time), Suicide risk (SR), Suicide risk in the past week (SR in the past week), Suicide risk at the most depressed time (SR at the most depressed time), The intensity of suicidal ideation (the intensity of SI), Adolescent Social Support Scale (ASSS), Thyroid stimulating hormone (TSH), Free triiodothyronine (FT3), Free thyroxine (FT4), Total cholesterol (TC), Triglyceride (TG), High-density lipoprotein (HDL), Low-density lipoprotein (LDL), Body mass index (BMI), Anterior cingulate cortex (ACC), Choline/creatine (Cho/Cr), Choline/N-acetyl aspartic acid (Cho/NAA), N-acetyl aspartic acid /creatine (NAA/Cr), Prefrontal cortex (PFC), Thalamus (Th).

## Data Availability

Not applicable.

## LIST OF ABBREVIATIONS

ACC: Anterior Cingulate Cortex
BMI: Body Mass Index
CDI: Children's Depression Inventory
ECT: Electroconvulsive Therapy
HDL: High-density Lipoprotein
HPT: Hypothalamic-pituitary-thyroid
LDL: Low-density Lipoprotein
MDD: Major Depressive Disorder
MRS: Magnetic Resonance Spectroscopy
NAA: N-acetyl Aspartic Acid
PFC: Prefrontal Cortex
SA: Suicide Attempt
TBS: Theta-burst Stimulation
TC: Total Cholesterol
TG: Triglyceride
TSH: Thyroid Stimulating Hormone
