# Supplementary material for: Metabolomic Changes in Major Depressive Disorder Adolescent Females with or without Suicide Attempts
Source: Curr Neuropharmacol. 2025 Jan 22;23(7):787–99. doi: 10.2174/1570159X23666250122093451 (PMC12163494; doi:10.2174/1570159X23666250122093451)
Supplement: Supplementary file 1 [file CN-23-7-787_SD1.pdf]

Supplementary Material

Metabolomic Changes in Major Depressive Disorder Adolescent Females with or without Suicide Attempts

Wei-Xi Deng[1,2,3], Xiao-Bo Liu[1,2], Tian Guo[1,2], Li-Fei Shang[1,2], Yi Li[1,2], Kuan Zeng[1,2,*] and Jing-Yi Long[1,2,*]

[1]Department of Radiology, Wuhan Mental Health Center, Wuhan, China; [2]Affiliated Wuhan Mental Health Center, Tongji Medical College of Huazhong University of Science and Technology, Wuhan, China; [3]Department of Psychiatry, The Second Affiliated Hospital and Yuying Children's Hospital of Wenzhou Medical University, Wenzhou, China

1875-6190/25 $65.00+.00 **© 2025 Bentham Science Publishers**

Table S1. Clinical and demographic characteristics for the three groups [$\bar{x}$±s/M(P25, P75)] or [n (%)].

Variable		MDD+SA (n =71)	MDD-SA（n=66）	HC（n=47）	H/Z/χ2	*P*	Contrast
**Sociodemographic**							
Age (years)		15(13,16)	15(13.75,16)	14(13,16)	3.594	0.166[a]	
Education (years)		8(7,9)	8(7,10)	7(6,10)	2.836	0.242[a]	
Disease course (months)		23(7,30)	14.5(5,26.5)	/	1.639	0.101[b]	
Current academic performance level	Unsatisfactory	15(21.13%)	10(15.15%)	2(4.26%)	11.375	0.077[c]	
	Modest	25(35.21%)	33(50.00%)	29(61.70%)			
	Good	26(36.62%)	19(28.79%)	12(25.53%)			
	Excellent	5(7.04%)	4(6.06%)	4(8.51%)			
Monthly disposable income	≦100	19(26.76%)	24(36.36%)	13(27.66%)	5.787	0.680[d]	
	100-500	29(40.85%)	26(39.39%)	15(31.91%)			
	500-1000	13(18.31%)	8(12.12%)	13(27.66%)			
	1000-2000	8(11.27%)	6(9.09%)	5(10.64%)			
	≧2000	2(2.82%)	2(3.03%)	1(2.13%)			
Family status	Living in harmony	4(5.63%)	8(12.12%)	14(29.79%)	28.070	<0.001[c]	
	The relationship is stable	32(45.07%)	42(63.64%)	27(57.45%)			
	The relationship is general	24(33.80%)	10(15.15%)	4(8.51%)			
	The relationship is bad	11(15.49%)	6(9.09%)	2(4.26%)			
Relationship with close contacts	very satisfactory	2(2.82%)	6(9.09%)	7(14.89%)	34.660	<0.001[d]	
	Be fairly satisfied	11(15.49%)	13(19.70%)	25(53.19%)			
	General relationship	38(53.52%)	33(50.00%)	13(27.66%)			
	Less satisfied	12(16.90%)	10(15.15%)	2(4.26%)			
	Be unsatisfied	8(11.27%)	4(6.06%)	0(0.00%)			
Satisfaction with school	very satisfactory	4(5.63%)	6(9.09%)	9(19.15%)	23.930	<0.001[c]	
	Be fairly satisfied	22(30.99%)	26(39.39%)	29(61.70%)			
	Less satisfied	29(40.85%)	24(36.36%)	6(12.77%)			
	Be unsatisfied	16(22.54%)	10(15.15%)	3(6.38%)			
Description of self-health status	Very health	5(7.04%)	12(18.18%)	14(29.79%)	37.706	<0.001[b]	
	Good	12(16.90%)	19(28.79%)	24(51.06%)			
	General	41(57.75%)	25(37.88%)	8(17.02%)			
	Poor	13(18.31%)	10(15.15%)	1(2.13%)			
Smoking	Yes	6(8.45%)	1(1.52%)	1(2.13%)	3.878	0.115[d]	
	No	65(91.55%)	65(98.48%)	46(97.87%)			
Alcohol consumption	Yes	8(11.27%)	3(4.55%)	2(4.26%)	2.690	0.290[d]	
	No	63(88.73%)	63(95.45%)	45(95.74%)			
**Clinical**							
CDI		34.35±7.70	27(19.75, 33)	15(8,17)	116.828	<0.001[a]	S>NS>N
SAS		61.73±12.22	54.09±13.80	/	-3.432	<0.001[e]	
SDS		69.07(60,77.5)	62.40(57.19, 69)	/	3.289	<0.001[b]	
BSI-CV	The scores for SI	24(21,27)	20(16,24)	14(11,20)	60.663	<0.001[a]	S>NS>N
	SI in the past week	10(8,13)	8(6,10)	6(5,8)	41.559	<0.001[a]	S>NS>N
	SI at the most depressed time	14(13,15)	12(9.75,14)	8(6,11)	60.764	<0.001[a]	S>NS>N
	SR	60(47,66)	40.5(35,52.25)	27(0,39)	76.036	<0.001[a]	S>NS>N
	SR in the past week	51.52(27.27,63.64)	19.70(6.06, 39.39)	0(-27.27,21.21)	61.311	<0.001[a]	S>NS>N
	SR at the most depressed time	75.76(66.67,84.85)	53.04(36.36,66.67)	21.21(-27.27,45.45)	81.238	<0.001[a]	S>NS>N
The intensity of SI		14.86±4.11	12(9.75,14.25)	/	4.042	<0.001[b]	/
Severity of SI	Non-SI	0(0.00%)	8(12.12%)	/	60.841	<0.001[d]	/
	Hope to die	0(0.00%)	4(6.06%)	/			
	Non-specific positive SI	3(4.23%)	9(13.63%)	/			
	No intention to take action	6(8.45%)	23(34.85%)	/			
	Intention to take action, but no specific plan.	20(28.17%)	18(27.27%)	/			
	Have specific plans	42(59.15%)	4(6.06%)	/			
ASLEC	Total	62.96±19.61	46.26±19.11	35(20, 69)	27.986	<0.001[a]	S>NS,N
	Interpersonal relationship	16(13, 19)	11.98±5.52	10.49±6.17	26.822	<0.001[a]	S>NS,N
	Study stress	13.48±5.25	12(7,14)	8.45±4.82	23.138	<0.001[a]	S>NS>N
	Penalty	14.07±6.72	8(5,12.25)	7(3,17)	23.811	<0.001[a]	S>NS,N
	Loss	4(1,9)	1.5(0,6)	3(1,8)	9.465	<0.009[a]	S>NS,N
	Healthy adaptation	7.82±3.77	5(3,7)	5(3,8)	17.672	<0.001[a]	S>NS,N
ASSS	Total	43.45±14.57	53(44.75,65)	64(52,78)	35.948	<0.001[a]	S<NS,N
	Subjective support	12(8,16)	16(12,20.25)	18.53±5.94	29.666	<0.001[a]	S<NS,N
	Objective support	17.41±6.16	21(18,24.25)	23(19,29)	24.109	<0.001[a]	S<NS,N
	Support utilization	13(9,16)	16(12,22.25)	21(17,26)	27.367	<0.001[a]	S<NS<N
a.Kruskal-Wallis H Test	b. Mann-Whitney U test	c. Pearson's chi-squared test		d.Fisher's exact test		e. two-sided t test	

Abbreviations: Healthy control (HC or N), Depression with suicide attempt (MDD+SA(S)), Depression without suicide attempt (MDD-SA(NS)), Children's Depression Inventory (CDI), Self-rating Anxiety Scale (SAS), Self-Rating Depression Scale (SDS), Scale for Suicide Ideation-Chinese Version (BSI-CV), The intensity of suicidal ideation (The intensity of SI), Severity of suicidal ideation (Severity of SI), Nonsuicidal ideation (Non-SI), Non-specific positive suicidal ideation (Non-specific positive SI), Adolescent Self-Rating Life Events Check List (ASLEC), Adolescent Social Support Scale (ASSS).

Table S2. Logistics analysis of SA in adolescents MDD patients.

	B	Standard error	*p*	OR	95% CI	
					Lower limit	Upper limit
**SR at the most depressed time**	0.038	0.017	0.025	1.039	1.005	1.074
**ASLEC**	0.036	0.014	0.008	1.037	1.009	1.064
**ASSS**	-0.040	0.019	0.035	0.960	0.925	0.997
**Severity of SI**	1.159	0.296	<.001	3.187	1.785	5.693
**Constant**	-6.909	1.898	<.001	0.001	/	/

Abbreviations*:* Severity of suicide ideation (Severity of SI), Adolescent Self-Rating Life Events Check List (ASLEC), Adolescent Social Support Scale (ASSS), Suicide risk at the most depressed time(SR at the most depressed time).

Table S3. Comparison of metabolic indexes [$\bar{x}$ ±s/M (P25, P75)].

Variable	MDD+SA	MDD-SA	HC	Z/t/χ2/H	*P*	Contrast
**Sample size**	56	45				
TSH(uIU/ml)	2.47(1.50,4.08)	2.31(1.77,3.43)	/	0.307	0.758[a]	
FT3(pg/ml)	2.95(2.70,3.33)	2.86±0.35	/	1.787	0.074[a]	
FT4(ng/dL)	1.19(1.09,1.38)	1.18(1.03,1.35)	/	0.673	0.501[a]	
**Sample size**	59	51	/			
TC(mmol/L)	4.01±0.61	3.9±0.76	/	-0.778	0.433[b]	
TC(mmol/L)	0.95(0.71,1.31)	0.91(0.66,1.20)	/	0.707	0.479[a]	
HDL(mmol/L)	1.21(1.09,1.46)	1.26±0.25	/	-0.492	0.623[a]	
LDL(mmol/L)	1.96±0.52	1.75(1.47,2.40)	/	1.037	0.300[a]	
BMI	20.14(17.97,22.76)	19.49(17.40,21.80)	/	0.986	0.324[a]	
**Sample size**	31	22	20			
ACC-Cho/Cr	1.01(0.94,1.14)	1.06±0.15	1.02(0.92,1.10)	0.388	0.824[d]	/
ACC-Cho/NAA	0.63(0.59,0.69)	0.65±0.62	0.66±0.06	0.987	0.611[d]	/
ACC-NAA/Cr	1.57(1.54,1.79)	1.65±0.17	1.54(1.46,1.75)	2.820	0.244[d]	/
PFC-Cho/Cr	0.96(0.89,1.04)	0.93±0.07	0.89±0.14	7.331	0.026[d]	S>N
PFC-Cho/NAA	0.50±0.04	0.47(0.46,0.51)	0.57±0.18	2.426	0.297[d]	/
PFC-NAA/Cr	1.96±0.19	1.92±0.14	1.68±0.42	7.401	0.001[e]	S, NS>N
Thalamus-Cho/Cr	0.95±0.10	0.98±0.15	0.97±0.08	0.477	0.623[e]	/
Thalamus-Cho/NAA	0.50±0.04	0.48±0.05	0.48(0.46,0.53)	1.588	0.452[c]	/
Thalamus-NAA/Cr	1.91±0.11	2(1.94,2.10)	1.98(1.86,2.06)	6.773	0.034[c]	S<NS

a.Kruskal-Wallis H Test, b.Two-sample t test, c.Fisher's exact test, d.Mann-Whitney U test
e.One-way analysis of variance

Abbreviations: Thyroid stimulating hormone (TSH), Free triiodothyronine (FT3), Free Thyroxine (FT4), Total cholesterol (TC), Triglyceride (TG), High-density lipoprotein (HDL), Low-density Lipoprotein (LDL), Body Mass Index (BMI), Anterior Cingulate Cortex (ACC), Choline/Creatine (Cho/Cr), Choline/N-acetyl aspartate (Cho/NAA), N-acetyl aspartate/Creatine (NAA/Cr), Prefrontal cortex (PFC).

Table S4. Correlation analysis of clinical features and metabolic indexes.

	TSH	FT3	FT4	BMI	TC	TG	HDL	LDL	ACC-Cho/Cr	ACC-Cho/NAA	ACC-NAA/Cr	PFC-Cho/Cr	PFC-Cho/NAA	PFC-NAA/Cr	Th-Cho/Cr	Th-Cho/NAA	Th-NAA/Cr
**CDI**	-0.06	0.12	-0.24	0.33	0.12	0.19	0.05	0.21	0.00	-0.09	0.06	-0.01	0.31	-0.27	0.10	0.22	-0.17
**BSI-CV**	-0.02	0.21	-0.05	0.42*	0.36*	0.16	0.21	0.31	0.12	-0.08	0.20	-0.01	0.28	-0.26	-0.06	0.16	-0.41*
**SI in the past week**	0.10	0.16	-0.19	0.34	0.32	0.12	0.08	0.51**	0.13	-0.14	0.22	-0.23	0.04	-0.30	-0.04	0.25	-0.43*
**SI at the most depressed time**	-0.05	0.21	0.07	0.42*	0.30	0.15	0.24	-0.04	-0.05	-0.04	0.04	0.20	0.39*	-0.12	-0.08	0.02	-0.33
**SR**	0.10	0.24	-0.06	0.43*	0.37*	0.35*	0.14	0.35*	0.12	-0.02	0.16	-0.11	0.08	-0.23	-0.03	0.23	-0.46**
**SR in the past week**	0.13	0.15	-0.08	0.43*	0.25	0.24	0.05	0.40*	0.10	-0.03	0.11	-0.10	0.12	-0.25	-0.02	0.28	-0.42*
**SR at the most depressed time**	0.05	0.27	0.01	0.36*	0.47**	0.41*	0.19	0.19	0.13	-0.04	0.23	-0.03	-0.01	-0.08	-0.05	0.14	-0.43*
**The intensity of SI**	0.02	0.13	0.08	0.35*	0.37*	0.32	0.09	0.20	-0.03	-0.04	0.01	0.08	0.25	-0.17	-0.07	0.09	-0.35*
**ASLEC**	-0.10	-0.18	-0.34	0.27	0.17	-0.07	0.24	0.08	-0.17	-0.12	-0.20	0.11	0.15	0.02	0.02	0.10	-0.18
**ASSS**	0.04	-0.23	0.03	-0.52**	-0.12	-0.20	-0.03	-0.24	-0.01	0.38*	-0.32	-0.15	-0.25	-0.02	-0.04	-0.20	0.23
**SAS**	0.01	0.06	-0.08	0.16	0.02	0.11	-0.10	0.21	0.11	0.08	0.05	-0.07	0.16	-0.28	0.00	0.10	-0.10
**SDS**	-0.06	-0.11	-0.27	0.08	0.25	-0.07	0.17	0.21	0.22	-0.03	0.26	-0.03	0.29	-0.29	0.11	0.15	-0.09
**TSH**				0.03	-0.13	0.35*	-0.49**	0.10	0.15	0.25	0.07	0.15	-0.32	0.36*	-0.39*	-0.29	-0.27
**FT3**				0.31	-0.18	0.25	-0.35*	0.18	0.18	0.09	0.19	0.08	-0.08	0.13	-0.31	-0.30	-0.19
**FT4**				0.03	-0.24	0.27	-0.09	-0.29	-0.03	-0.01	0.01	0.13	-0.15	0.24	-0.42*	-0.33	-0.21
**BMI**									0.00	-0.06	0.09	0.13	0.19	0.08	0.07	0.13	-0.23
**TC**									0.07	-0.04	0.04	-0.07	0.03	-0.13	0.05	0.24	-0.30
**TG**									0.13	0.06	0.16	0.20	0.03	0.14	-0.25	-0.03	-0.33
**HDL**									-0.09	-0.12	-0.08	-0.19	0.15	-0.28	0.23	0.28	0.03
**LDL**									0.22	-0.06	0.22	-0.11	0.00	-0.13	0.07	0.14	-0.16

p <0.05, p* *<0.01;*
Abbreviations: Scale for Suicide Ideation-Chinese Version (BSI-CV), suicide ideation in the past week (SI in the past week), suicide ideation at the most depressed time (SI at the most depressed time), Suicide risk (SR), suicide risk in the past week (SR in the past week), suicide risk at the most depressed time (SR at the most depressed time), The intensity suicide ideation (The intensity of SI), Adolescent Social Support Scale (ASSS), Thyroid stimulating hormone (TSH), Free triiodothyronine (FT3), Free thyroxine (FT4), Total cholesterol (TC), Triglyceride (TG), High-density lipoprotein (HDL), Low-density Lipoprotein (LDL), Body Mass Index (BMI), Anterior Cingulate Cortex (ACC), Choline/Creatine (Cho/Cr), Choline/N-acetyl aspartic acid (Cho/NAA), N-acetyl aspartic acid/Creatine (NAA/Cr), Prefrontal cortex (PFC), Thalamus (Th).
